# Personalised bactericidal combination regimens against carbapenem-resistant *Pseudomonas aeruginosa*

**DOI:** 10.1038/s43856-025-01022-2

**Published:** 2025-08-05

**Authors:** Jocelyn Qimin Teo, Jing Heng Toh, Hong Yi Chang, Si Hui Tan, Jayden Jun-Yuan Ho, Zhi Wei Ong, Winnie Lee, Yen Ee Tan, Tse Hua Nicholas Wong, Shimin Jasmine Chung, Thuan Tong Tan, Tze Peng Lim, Andrea Lay-Hoon Kwa

**Affiliations:** 1 Division of Pharmacy, Singapore General Hospital, Singapore, Singapore; 2 SingHealth Duke-NUS Pathology Academic Clinical Programme, Singapore, Singapore; 3 Department of Pharmacy, Khoo Teck Puat Hospital, Singapore, Singapore; 4 Department of Clinical Translational Research, Singapore General Hospital, Singapore, Singapore; 5 Department of Microbiology, Singapore General Hospital, Singapore, Singapore; 6 Department of Infectious Diseases, Singapore General Hospital, Singapore, Singapore; 7 SingHealth Duke-NUS Medicine Academic Clinical Programme, Singapore, Singapore; 8 Emerging Infectious Diseases, Duke-National University of Singapore Medical School, Singapore, Singapore

**Keywords:** Bacterial infection, Antimicrobial resistance

## Abstract

**Background:**

Limited treatment options are available for carbapenem-resistant *Pseudomonas aeruginosa* (CRPA), especially those harbouring carbapenemases. Given the variability in effectiveness of antibiotic combinations, in vitro test-guided antibiotic therapies have been suggested to improve the outcomes of patients receiving combination therapy. We investigated the in vitro bactericidal activities of various combinations against CRPA and described our experience of using a personalised test-guided approach in managing these difficult-to-treat infections.

**Methods:**

In vitro antibiotic combination tests (*i*ACTs) comprising up to 180 unique combinations at clinically relevant unbound concentrations were performed for CRPA isolates collected for a monocentric prospective cohort study conducted between 2017 and 2022.

**Results:**

In vitro activities of antibiotic combinations are highly strain-specific. Polymyxin-containing combinations exhibit the highest bactericidal activity [454/497 (91%) isolate-combination pairs] against 66 isolates tested. Most of the CRPA produce carbapenemases (73%). Polymyxin-sparing combinations exhibiting >60% bactericidal activity include fosfomycin + aztreonam (40/58) and fosfomycin + cefepime (37/58). We show good outcomes in the 42 patients who eventually received at least 72 h of personalised *i*ACT-guided therapy [end-of-treatment clinical response rate: 93%; 30-day all-cause mortality: 2%]. We observe microbiological eradication for all bloodstream infections, but 13% of patients get reinfected with CRPA subsequently.

**Conclusions:**

Polymyxin-containing combinations exhibit the highest bactericidal activity against CRPA, while fosfomycin +  aztreonam or fosfomycin + cefepime represent promising polymyxin-sparing options. A personalised in vitro test-guided treatment approach may be feasible for managing *P. aeruginosa* infections, particularly those involving carbapenemase production and/or difficult-to-treat phenotypes.

## Plain language summary

Doctors face difficulties treating serious infections caused by carbapenem-resistant *Pseudomonas aeruginosa* (CRPA), a bacterium that is difficult to treat with common antibiotics. We examined how effective various antibiotic combinations are against CRPA using antibiotic combination tests in the lab. The tests revealed variable effectiveness of the antibiotic combinations and were used to tailor treatment to each patient. Patients receiving these personalised regimens showed improved recovery, infection clearance, and 30-day survival. This suggests that using lab test-guided, personalised antibiotic combinations may help doctors to treat difficult CRPA infections more successfully, especially when standard treatments fail.

## Abstract (web summary)

Teo, Toh et al., evaluate the in vitro bactericidal activities of antibiotic combinations and the outcomes of a tailored test-guided treatment approach for carbapenem-resistant *Pseudomonas aeruginosa* infections. They observe positive clinical and microbiological outcomes, suggesting the approach’s feasibility for treating infections where other treatment options are scarce.

## Introduction

The rapid emergence of carbapenem-resistant (CR) *Pseudomonas aeruginosa* (PA) is a serious public health threat^1,2^. They are difficult to treat due to limited treatment options and, therefore associated with serious infections with high mortality rates^3,4^. Whilst carbapenemase production in CRPA is less frequently observed in certain geographic regions such as the United States, the prevalence of carbapenemase-producing CRPA isolated from other areas such as Singapore, Australia, and South/Central America is high^5^. The utility of newer agents such as ceftazidime-avibactam and ceftolozane-tazobactam is limited by the production of carbapenemases such as metallo-*β*-lactamases. Resistance to these new agents has also been reported^6^. Often, this leaves clinicians with antibiotic combination therapy as the remaining option in the management of such difficult-to-treat (DTR) CRPA infections.

Unfortunately, there is still a dearth of evidence in antibiotic combination choices for CRPA infections, especially those caused by carbapenemase-producing CRPA. In vitro studies have been performed to identify optimal combination regimens for CRPA with varied resistance mechanisms. Interactions observed in vitro are highly variable, unpredictable, and may range from antagonism to high rates of synergy^7,8^. High synergy rates and/or improved bactericidal activities have been observed primarily in carbapenem-aminoglycoside combinations, which are often poorly tolerated in practice due to aminoglycoside-associated nephrotoxicity^9^. Furthermore, well-established antibiotic combinations involving newer agents effective against metallo-*β*-lactamase (MBL)-producing Enterobacterales, such as ceftazidime-avibactam + aztreonam, cannot be extrapolated to MBL-producing CRPA^10^.

Given the variability in effectiveness of antibiotic combinations, in vitro test-guided antibiotic therapies have been suggested to improve outcomes of patients receiving combination therapy; however, the clinical relevance of this strategy remains controversial^11,12^. Moreover, most of these tests rely on determining in vitro synergy, which may not necessarily translate to improved bactericidal killing^13^. Here, we aim to evaluate the in vitro bactericidal activities of antibiotic combinations against CRPA and the utility of a personalised in vitro-guided antibiotic combination therapy clinical service in the treatment of these infections.

Patients receiving bactericidal combinations tailored to their individual characteristics and the ability of the agents to penetrate the infection site have good clinical and microbiological response and 30-day survival. Our results support such a test-guided personalised approach in treating DTR CRPA.

## Methods

### Study design

This was a single-centre prospective cohort study conducted in Singapore General Hospital (SGH), a 1800-bedded public acute care tertiary hospital. Patients ≥21 years old admitted between October 2017 and October 2022 with positive CRPA clinical cultures were prospectively identified from the institution’s microbiology laboratory. In vitro antibiotic combination tests (*i*ACTs) were conducted on CRPA isolates deemed to be implicated in infections by the attending infectious diseases (ID) physicians. The research and its study protocol were approved by the SingHealth Centralised Institutional Review Board (Reference number: 2017/2047). Patients’ consent was sought prior to *i*ACT commencement.

### Microbiological methods

The *i*ACT workflow and methodology have been published previously^14^. The *i*ACT was carried out in clear 96-well microtitre plates containing 100 μL of various antibiotics in single, two-drug, and three-drug combinations at clinically relevant unbound concentrations in each well. Up to 180 different antibiotic combinations were tested each time. These panels were incubated for 24 h at 35–37 °C and assessed for the presence or absence of visible growth in each well. The contents of each clear well were then sampled for bacterial enumeration to determine bactericidal activity (≥3 log_10_CFU/mL reduction of the initial bacterial inoculum at 24 h). Minimum inhibitory concentrations were determined using commercial microbroth dilution panels (Trek Diagnostics, East Grinstead, UK), except for fosfomycin, which was tested using antibiotic gradient test strips (bioMérieux, Durham, USA) and interpreted using the Clinical and Laboratory Standards Institute clinical breakpoints^15^. Carbapenem resistance was defined as non-susceptibility to meropenem/imipenem/doripenem. Genomic profiles of isolates were characterised using paired-end whole-genome sequencing (WGS) using MiSeq/HiSeq/Novaseq systems (Illumina Inc., CA, USA)^16^. Acquired resistance genes and chromosomal variants were identified using the NCBI-AMRFinderPlus database^17^.

The *i*ACT clinical service workflow is depicted in Supplementary Fig. 1. An ID specialty-trained pharmacist reviewed the patient’s clinical details and interpreted the test results. At 24 h, if the initial empiric regimen was not at least bacteriostatic in vitro (indicated by visible growth in the test well), the pharmacist-in-charge made a preliminary recommendation to change the antibiotic combination. At 48 h, the pharmacist-in-charge identified all available bactericidal combinations and prepared a final report detailing the treatment recommendations for the attending ID physician (Supplementary Fig. 2). The treatment recommendations included personalised bactericidal combinations and corresponding dosing regimens tailored according to each patient’s characteristics and the ability of individual antibiotics to penetrate the infection site. In select cases, dosing individualisation was provided where therapeutic drug monitoring is available. After the initiation of *i*ACT-guided treatment, the ID pharmacist is involved in the monitoring of the safety and efficacy of the recommended regimen and provides *i*ACT-guided therapeutic plan adjustments where necessary.

### Clinical data collection and outcomes assessment

Patients who received *i*ACT-guided bactericidal regimens for at least 72 h from the time of *i*ACT initiation were assessed for clinical outcomes. All clinical data were collected from the electronic medical records and documented in a structured data collection form. Patients were followed up till discharge or death, whichever occurred earlier. The clinical and microbiological outcomes included 30-day all-cause mortality, 30-day infection-related mortality, end-of-therapy clinical response, end-of-therapy microbiological eradication, and adverse drug reactions (ADRs) associated with *i*ACT-guided therapies. Clinical response was defined as partial or complete resolution of infective signs/symptoms and/or laboratory examination (e.g. vital signs, inflammatory markers, etc) at the end of therapy. Microbiological eradication was determined only for CRPA bacteraemia and defined as CRPA clearance for two consecutive sets of blood cultures by the end of therapy.

### Reporting summary

Further information on research design is available in the Nature Portfolio Reporting Summary linked to this article.

## Results

### Characteristics of CRPA

Sixty-six CRPA clinical isolates were evaluated for in vitro bactericidal activity. The majority of CRPA were isolated from the blood (16, 24%), the respiratory tract (13, 20%), the genitourinary tract (11, 17%), and skin/soft tissue (12, 18%). The antibiotic susceptibilities are displayed in Table 1 (see Supplementary Data 1 for detailed phenotypic and genotypic characteristics of the individual isolates). Fifty-seven (86%) isolates were classified as DTR^18^. Susceptibilities to the new *β*-lactam/*β*-lactamase inhibitors were also low (ceftolozane-tazobactam-8%, ceftazidime-avibactam-14%).

**Table 1:** Antimicrobial susceptibility patterns of 66 *i*ACT isolates

Antibiotic	% *S*	% *I*/SDD	% *R*	MIC data (mg L^−1^)		
				MIC_50_	MIC_90_	Range
Doripenem	0	5	95	≥64	≥64	4 to ≥64
Imipenem	3	2	95	≥64	≥64	2 to ≥64
Meropenem	2	3	95	≥64	≥64	1 to ≥64
Aztreonam	6	40	54	32	≥64	4 to ≥64
Cefepime	1	5	94	≥128	≥128	4 to ≥128
Piperacillin/Tazobactam	1	5	94	≥128/4	≥128/4	16/4 to ≥128/4
Levofloxacin	3	2	95	≥32	≥32	≤0.5 to ≥32
Amikacin	33	6	61	64	≥128	≤4 to ≥128
Polymyxin B	-	97	3	1	2	≤0.25 to 4
Ceftazidime/Avibactam	14	-	86	≥128/4	≥128/4	4/4 to ≥128/4
Ceftolozane/Tazobactam	8	4	88	≥128/4	≥128/4	0.5/4 to ≥128/4
Fosfomycin	-	-	-	64	256	0.25 to ≥2048

*I* intermediate, *S* susceptible, *SDD* susceptible dose-dependent, *R* resistant.

Similar to the molecular epidemiology of CRPA in general in our institution, isolates included in this study were highly diverse [20 different sequence types (STs)], although most belonged to the high-risk clonal groups ST308 (32, 49%) and ST235 (14, 21%). Carbapenemases were detected in 48 (73%) isolates, with the predominance of New Delhi metallo-*β*-lactamase (NDM) enzymes (32/48, 67%). Additionally, CRPA harboured a large array of acquired antibiotic resistance genes, frequently those targeting resistance to aminoglycosides, quinolones, and sulphonamides. *β*-lactamase genes were also detected. Although we did not study the mutational genomes of CRPA here, our previous studies suggest that the local CRPA has the propensity to acquire diverse chromosomal mutations, potentially leading to diverse CRPA phenotypes^6^.

### Bactericidal combinations against CRPA isolates

Here we present the in vitro results of selected combinations which are most commonly employed at SGH (Table 2). Bactericidal activity was demonstrated in 885 of the 1579 (56%) isolate-combination pairs that were evaluated. Polymyxin-containing combinations resulted in the highest bactericidal activity [454/497 (91%) isolate-combination pairs evaluated]. Bactericidal activities of individual polymyxin-containing combinations ranged from 86 to 97%.

**Table 2:** Bactericidal activities of selected combinations against 66 *i*ACT isolates

		Number of isolates tested	Number with bactericidal activity	% bactericidal
Polymyxin B	Meropenem	66	63	95
	Aztreonam	66	61	92
	Cefepime	66	58	88
	Piperacillin-tazobactam	66	57	86
	Ceftolozane-tazobactam	55	50	91
	Ceftazidime-avibactam	54	49	91
	Levofloxacin	66	60	91
	Fosfomycin	58	56	97
Fosfomycin	Meropenem	58	32	55
	Aztreonam	58	40	69
	Cefepime	58	37	64
	Piperacillin-tazobactam	58	25	43
	Ceftolozane-tazobactam	55	28	51
	Ceftazidime-avibactam	55	26	47
	Levofloxacin	58	28	48
Aztreonam	Meropenem	66	19	29
	Cefepime	66	32	48
	Piperacillin-tazobactam	66	21	32
	Ceftolozane-tazobactam	55	26	47
	Ceftazidime-avibactam	55	25	45
	Levofloxacin	66	30	45
Levofloxacin	Meropenem	66	11	17
	Cefepime	66	15	23
	Piperacillin-tazobactam	66	8	12
	Ceftolozane-tazobactam	55	14	25
	Ceftazidime-avibactam	55	14	25

Compared to polymyxin-containing combinations, polymyxin-sparing combinations were bactericidal against a lower proportion of isolates. Excluding polymyxin-containing combinations, bactericidal activity was observed in 216/400 (54%), 193/432 (45%), and 120/432 (28%) fosfomycin-containing, aztreonam-containing, and levofloxacin-containing combination-isolate pairs, respectively. Among these, only the following antibiotic pairs were bactericidal against >50% of the isolates: fosfomycin + aztreonam (40/58, 69%), fosfomycin + cefepime (37/58, 64%), fosfomycin + meropenem (32/58, 55%), and fosfomycin + ceftolozane-tabobactam (28/55, 51%). Notably, ceftazidime-avibactam + aztreonam, a combination increasingly employed against carbapenem-resistant Enterobacterales, was bactericidal only against 25/55 (45%) isolates.

### Clinical outcomes

Out of the 66 isolates, 42 cases were evaluated for clinical outcomes. Twenty-four cases were excluded from clinical analyses due to the following reasons: demised prior to *i*ACT completion or receipt of at least 72 h of *i*ACT-guided therapy (*n* = 7); concomitant carbapenem-resistant organisms (non-pseudomonal) (*n* = 5); colonisation not requiring antimicrobial therapy (*n* = 3); consecutive isolates from same patients (*n* = 3). In addition, *i*ACT-guided therapy recommendations were not taken up by the ID physicians in 6 (9%) cases—they elected to utilise monotherapy/non-bactericidal combination therapy or wound care only, as these patients had already clinically improved at the time of *i*ACT results availability. It was unlikely that CRPA was the implicated organism in the infection (See Supplementary Data 2 for details).

**Fig. 1:**
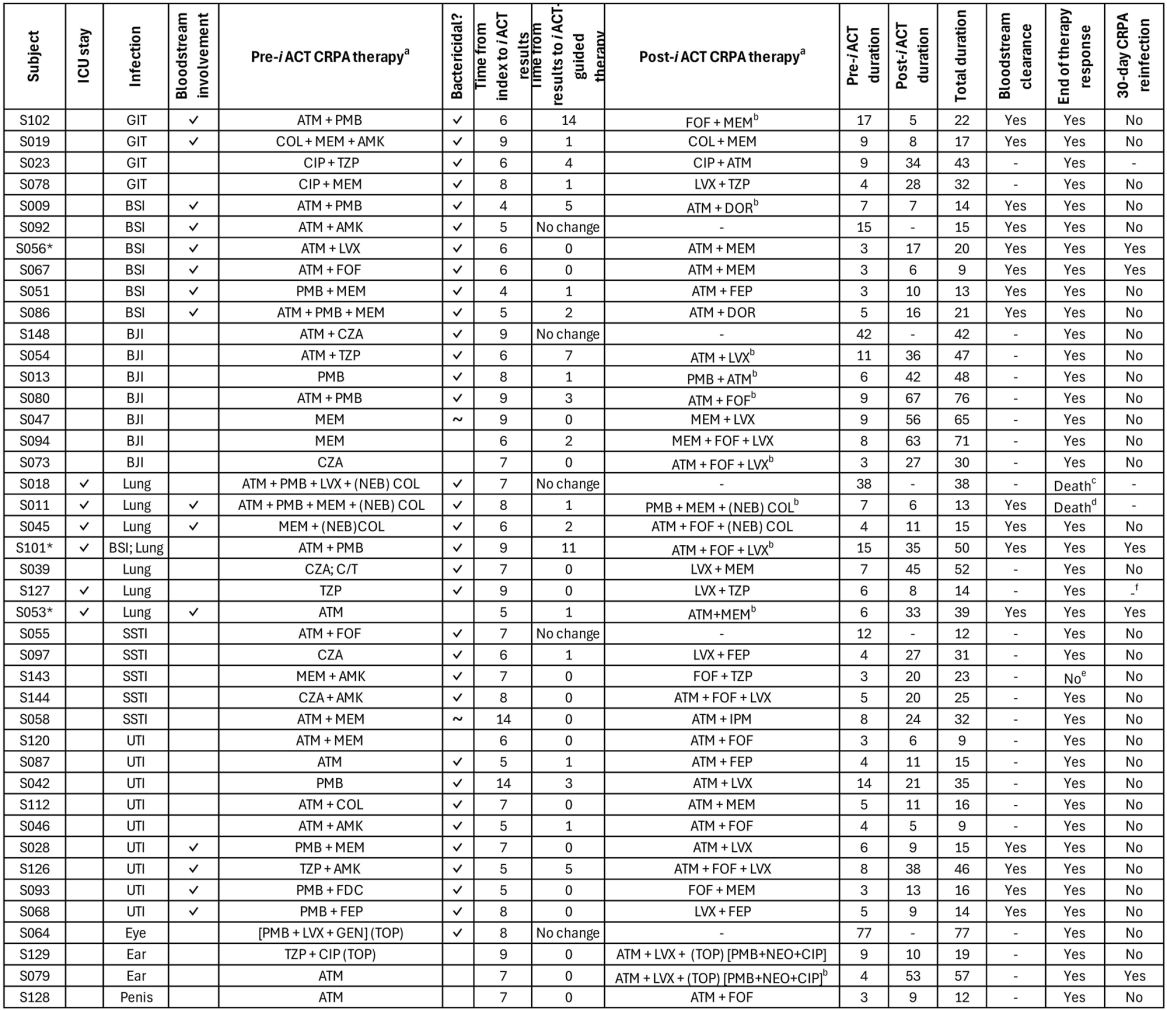
Treatment and outcomes of 42 clinically evaluable cases. *Two *i*ACTs conducted for these cases. “-” denotes non-applicable/non-evaluable. “~” denotes non-bactericidal regimens which are at least bacteriostatic. ^a^All agents listed are administered parenterally unless otherwise stated. ^b^Subsequent therapy changes are documented in Supplementary Data 4. ^c^Antimicrobials were withdrawn after 45 days in this patient with a background of lung adenocarcinoma afflicted with necrotising ventilator-associated pneumonia (concomitant fungal pneumonia) when she was transitioned to comfort measures. Patient demised a day after withdrawal of care. Lower respiratory tract/pleural cultures were negative prior to antibiotics withdrawal. ^d^Patient demised 6 days after initiating *i*ACT-guided therapy due to disseminated Candida/Aspergillus/Strongyloides/CRPA infections. ^e^Antibiotics were discontinued after 36 days since the patient developed neutropenia and his foot wound was not improving without source control. Subsequently, the patient underwent a below-knee amputation, and his wound healed well thereafter without antibiotics. ^f^Loss to follow-up as a foreign patient was discharged to a hospital in her home country. BSI primary bloodstream infection/sepsis unknown source, BJI bone and joint infection, GIT intra-abdominal/gastrointestinal tract infection, SSTI skin and soft tissue infection, UTI genitourinary tract infection, NEB nebulised, TOP topical, AMK amikacin, ATM aztreonam, CAZ ceftazidime, CIP ciproflaxacin, COL colistin, C/T ceftolozane/tazobactam, CZA ceftazidime-avibactam, FDC cefiderocol, FEP cefepime, FOF fosfomycin, GEN gentamicin, LVX levofloxacin, MEM meropenem, NEO neomycin, PMB polymyxin B, TZP piperacillin-tazobactam.

The patient characteristics and treatment characteristics are summarised in Table 3 and Fig. 1, respectively (see also Supplementary Data 3 for source data of Fig. 1). The median (IQR) total duration of CRPA-targeted therapy was 23 (26) days. Prior to the availability of *i*ACT results, 12 (29%) patients received monotherapy, 25 (60%) received two antibiotics, while the remaining were treated with three agents. These regimens frequently comprised aztreonam and/or polymyxins (30/42, 71%) to which most isolates retained intermediate susceptibilities compared to the other antibiotics (aztreonam MIC range: 4 –$\ge$64 mg/L, 55% susceptible/intermediate; polymyxin MIC range: ≤0.25–4 mg/L, 95% intermediate).

**Table 3:** Baseline demographics and clinical characteristics of 42 clinically evaluable cases

Characteristics	*N* = 42
Age, years (mean ± SD)	60.5 ± 14.2
Male	26 (61.9)
Body (mean ± SD) mass index, kg/m^2^	22.4 ± 4.8
Selected co-morbidities	
Diabetes	16 (38)
Chronic kidney disease	9 (21)
Solid and haematological malignancy	20 (48)
Immunocompromised status	10 (24)
Charlson comorbidity index, median (IQR)	5 (4)
SOFA score, median (IQR)	1 (3)
0–3	32 (76)
4–5	6 (14)
≥6	4 (10)
Intensive care unit at time of CRPA isolation	6 (14)
Renal replacement therapy	6 (14)
Type of infection	
Genitourinary tract	9 (21)
Bone and joint	7 (17)
Sepsis/primary bloodstream	6 (14)
Respiratory	6 (14)
Skin & soft tissue/surgical site	5 (12)
Intra-abdominal	4 (10)
Others^a^	5 (12)
Bloodstream involvement	16 (38)
Concomitant infections	28 (67)

Data presented as *n* (%) unless otherwise stated.

*CRPA* carbapenem-resistant Pseudomonas aeruginosa, *SOFA* sequential organ failure assessment.

^a^Include infections from eye (*n* = 1), ear (*n* = 2), penile prosthesis (*n* = 1), line infection which progressed to a lung infection (*n* = 1).

The median (range) time from index date to *i*ACT test was 7 (4–14) days. Bactericidal activity was demonstrated in 33 (79%) pre-*i*ACT regimens upon testing. Non-bactericidal pre-*i*ACT regimens were composed mostly of single agents. Combinations which were non-bactericidal included aztreonam + meropenem (*n* = 2) and piperacillin-tazobactam + ciprofloxacin (*n* = 1). Only 2 of 9 non-bactericidal regimens were at least bacteriostatic ( <3 log_10_CFU/mL reduction of the initial bacterial inoculum at 24 h).

Only five (12%) patients were maintained on the initial CRPA-targeted regimens prescribed by the attending ID physician throughout the entire treatment duration after *i*ACT. Aside from cases that were receiving non-bactericidal regimens, therapy changes were also effected to ensure that the eventual selected combination regimens were highly probable in achieving adequate concentrations at the infection sites. Other reasons for therapy modifications include: de-escalation from $\ge$3 agents; slow or non-response with pre-*i*ACT regimens; avoidance/development of adverse reactions (especially with polymyxin B regimens); cost and administration issues (e.g. require administration at the outpatient antibiotic clinic).

Individualised *i*ACT-guided antibiotic combinations commonly employ a polymyxin-sparing approach to avoid potential side effects associated with the prolonged use. Aztreonam was often included in the combination regimen (30/42, 71%), frequently with fosfomycin as the partner agent (11/30, 35%). As a result, intravenous (IV) polymyxin usage was reduced from 15 (36%) cases pre-*i*ACT to 4 (10%) cases post-*i*ACT. IV polymyxin use was retained in these cases because clinicians favoured continuing patients on these regimens where clinical improvement was already observed. Chronic kidney disease was absent in these cases, although 2 patients were experiencing acute kidney injury due to sepsis and were receiving continuous renal replacement therapy at the time of IV polymyxin administration.

Most patients were maintained on a single regimen post-*i*ACT, however, 10 cases required adjustments to their regimens in view of the complicated and dynamic infection episodes encountered. Reasons for therapy adjustments are documented in Supplementary Data 4 and include non-improvement, new septic episodes requiring broader coverage involving pathogens other than CRPA, and adverse drug reactions.

Overall, the end-of-therapy clinical response rate was 93% (39/42). Of the three non-responding cases, thirty-day all-cause mortality was observed only in one (2%) patient (Case S011) who demised 6 days after initiation of *i*ACT due to disseminated polymicrobial (CRPA, *Candida*, *Aspergillus,* and *Strongyloides*) infections involving the bloodstream and lungs. Microbiological eradication was observed in all patients with positive blood cultures by the end of therapy.

CRPA reinfections were observed within 30 days from the end of therapy in 5 of 38 (13%) of evaluable patients. WGS of subsequent strains which were available indicated that strains implicated in these recurrent infections were of similar STs and antimicrobial resistance profiles as the index strains. However, the initial *i*ACT regimens were found to be non-bactericidal in 3 cases where *i*ACTs were repeated. Interestingly, aztreonam + meropenem was the *i*ACT regimen used for treatment in 2 of these cases. Recurrent cases primarily occurred in patients with a risk of re-infection, such as those with persistent colonisation/reservoirs, e.g. ventilators/lines, chronic wounds, or difficult-to-eradicate infection sources, compounded by poor overall health and immune function.

*i*ACT regimens were mostly tolerable—ADRs attributed to antibiotics occurred in 12 (29%) cases. ADR types included drug exanthem (*n* = 1), diarrhoea (*n* = 1), hyperpigmentation (*n* = 1), neutropenia (*n* = 2), electrolyte derangement (*n* = 3), and neurotoxicity (e.g. confusion, tremors/myoclonic jerks, parasthesia, loss of sensation) (*n* = 6). Discontinuation of the suspected offending agents (e.g. discontinuation of all antibiotics, temporary suspension of offending agents, switching to alternative bactericidal regimens) was required in all except one case. Eventual resolution of symptoms was observed and did not impact outcomes, as the majority of the patients (10/12, 83%) responded clinically at the end of the combination therapy.

## Discussion

*P. aeruginosa*, especially those with CR/DTR resistance phenotypes, remains one of the most challenging pathogens to treat globally. The high genome plasticity of the *P. aeruginosa* complicates the management, necessitating contemporary evidence to support treatment practices for this constantly evolving pathogen^19^. Singapore’s status as an international travel and medical hub has seen the import and dissemination of antimicrobial resistance to the country. For this reason, the local CRPA epidemiology encompasses well-known epidemic multi-drug-resistant clones amidst a diverse pool of non-epidemic lineages. Here, we identified potential treatment options for the management of a genomically diverse CRPA cohort, primarily composed of carbapenemase-producing strains, using *i*ACT. Additionally, we shared our real-world clinical experience in treating these challenging infections with a personalised approach.

Based on *i*ACT results, polymyxin-containing combinations exhibited the highest bactericidal rates. Compared to the other test agents, where the agent’s MIC far exceeds the corresponding simulated test concentration, polymyxin MICs observed in CRPA were lower than or equivalent to the simulated concentration (2 mg/L) utilised in *i*ACT^20^, increasing the probabilities of enhanced bacterial killing when combined with a synergistic partner agent. Therefore, a polymyxin-containing combination might be a reasonable empiric combination prior to the availability of *i*ACT results or where *i*ACT is not available. Unfortunately, most patients afflicted with CRPA infections tend to have relative or absolute contraindications to polymyxins or are unable to tolerate the regimen for the entire treatment duration, which is often prolonged.

Alternative regimens identified in this study included fosfomycin-containing combinations. Specifically, fosfomycin + aztreonam was the polymyxin-sparing regimen that displayed the highest bactericidal rates in our study. This may be related to the epidemiology of CRPA in Singapore, where MBL-producing *P. aeruginosa* are encountered frequently. Aztreonam is not appreciably hydrolysed by MBLs, owing to poor and unproductive binding with the enzymes^21^. The addition of fosfomycin here exerted an additive/synergistic effect, resulting in bactericidal kill. This combination has also shown promise in various other studies where the aztreonam + fosfomycin combination demonstrated collective synergistic/additive rates above 80%, even restoring aztreonam susceptibility for some *P. aeruginosa*^22–24^.

Given fosfomycin’s and aztreonam’s wide tissue penetration, regimens containing this combination with/without levofloxacin were the fosfomycin-based regimen of choice targeting various infections in this study. IV fosfomycin disodium 8 g every 8 h was the most commonly employed dose in our patient cohort. Unfortunately, this dosing regimen came with the propensity for electrolyte derangement, particularly hypernatremia and hypokalemia, warranting closer monitoring. A good clinical response was observed in most cases utilising the fosfomycin + aztreonam combination in our study. The encouraging results both in vitro and in vivo should prompt further investigation of this combination, especially for the management of MBL-producing CRPA in general.

Apart from polymyxin-containing regimens that were able to target a large proportion of the isolates tested, *i*ACT results suggested that the in vitro activities of antibiotic combinations are highly strain-specific and unpredictable. Corresponding to this strain specificity, we observed that some antibiotic regimens utilised prior to *i*ACT were found to be non-bactericidal (or even non-inhibitory) upon in vitro testing. These antibiotic regimens were empirically selected by the treating physicians based on previous experience and/or anecdotal reports combined with routine antibiotic susceptibility testing results. Prolonged use of these inactive regimens could potentially lead to eventual antibiotic resistance or therapeutic failure^25,26^.

The *i*ACT service provided several benefits to the treatment of CRPA infections. All patients were eventually treated with bactericidal combinations with optimal pharmacokinetic/pharmacodynamic profiles, leading to a high proportion of patients with clinical response at the end of therapy. The role of a clinical ID pharmacist in this service was critical in therapy optimisation through individualising the antibiotic combination(s) according to patients’ clinical status, co-infections, and co-morbidities with guidance from the *i*ACT results. CRPA infections are often complicated, requiring prolonged therapy durations. In our experience, patients frequently have complex comorbid conditions resulting in turbulent and protracted infection courses. With the *i*ACT service, the patients are monitored throughout their infection episodes, allowing timely personalised therapy optimisation in response to dynamic changes during the infection course (e.g. organ function changes, adverse drug reactions, concomitant infections, etc). Furthermore, the *i*ACT encompassed >100 different combinations, greatly enhancing the treatment availability and flexibility in responding to these changes. This is accompanied by therapeutic drug monitoring, where available, to ensure that dose adjustments result in adequate antibiotic levels at infection sites.

Simultaneous testing of multiple varied combinations has also allowed the majority of patients to be treated with polymyxin-sparing regimens. Polymyxins are generally disfavoured by clinicians in view of the well-known toxicity profiles of the agent in our cohort and its poor tissue penetration^27,28^. Prolonged treatment durations exceeding 2 weeks, particularly in those with poor underlying kidney function, increase the risk of polymyxin-associated adverse drug reactions^29^. In this study, *i*ACT has allowed physicians to minimise polymyxin duration with the use of bactericidal non-polymyxin-containing regimens where activity was confirmed by the test, resulting in the avoidance of adverse drug reactions without compromising the clinical outcomes.

Combination therapy has been used as a tool for the prevention or delay of the emergence of resistance^30^. Although initial clinical response was observed in the majority of the patients, the CRPA re-infection rate was 13%, suggesting the potential emergence of resistance. *P. aeruginosa* is known to adapt readily to the host environment and can result in persistent colonisation, especially in biofilm-producing strains, leading to recurrent infections^31^. While antibiotic therapy forms the cornerstone of infection management, inadequate source control and host immune function are integral factors for successful treatment. Poor antibiotic penetration and slow growth within biofilms that may have formed on indwelling devices and in chronic wounds may have impaired both the killing effect of the aggressive combination antibiotic therapy administered, leading to the emergence of resistant phenotypes. This is further complicated by the compromised immune status as seen in several of the cases here. Resistant *P. aeruginosa* infections remain challenging to manage and may be refractory to even active combination therapy, especially in immunocompromised individuals with indwelling devices/chronic wounds, which can serve as potential sources of recurrent infection. Without the complete removal of such reservoirs, combination therapy can at best suppress the infection temporarily in such a scenario.

The *i*ACT-guided personalised treatment strategy is not without challenges. Current in vitro testing methods rely on viable cell counting, which requires a minimum of 48 h from the time of isolate receipt at the pharmacy laboratory. This is compounded by an inherent time delay involved in pathogen isolation and identification, which may lead to considerable delay in the time of receipt of *i*ACT therapy. Hence, due to current limitations of *i*ACT, such a strategy may be of limited benefit to critically-ill patients who require immediate active therapy. Nevertheless, the data generated in this study have identified selected combinations with a high proportion of bactericidal activity against our local CRPA clones, which could be used to inform the empiric treatment of severely ill patients while awaiting test results. This treatment strategy is also relatively resource-intensive and requires skilled laboratory personnel and specialist-trained pharmacists in the conduct and interpretation of the test. Therefore, the cost and resources associated with this approach may limit its access.

Although the study had multiple promising findings, it had several limitations. Firstly, this was a non-controlled study with recognised inherent biases and a small sample size. It is uncertain if patients receiving *i*ACT-guided personalised treatment fared better compared to those receiving unguided therapy. The favourable clinical outcomes we observed in this study could also be influenced by confounders, including disease severity and host factors such as co-morbidities/immune status and availability of non-antibiotic interventions. In addition, *P. aeruginosa* can exist as commensals (e.g. on skin, urine, airways), making it difficult to distinguish between colonisation and infection in febrile patients with polymicrobial cultures. At the time of *i*ACT initiation, treatment targeting the implicated bacteria was initiated, leading to clinical improvement due to the susceptibility of the bacteria to the chosen treatment. When *i*ACT results were available, the clinicians deemed *P. aeruginosa* to bear little clinical significance after assessment, choosing to manage the patients conservatively or continuing the therapy which were not active against CRPA. Test-guided treatment plans were reserved for clinical deterioration. Bias could have been introduced inadvertently through the exclusion of such cases from the outcomes analyses. Furthermore, the exclusion of patients who were unable to receive *i*ACT-guided therapy, such as those who died shortly after test initiation, from the analyses could have inflated the perceived effectiveness of the test. However, the poor outcomes observed in these patients were mostly related to underlying co-morbidities (e.g. malignancies) and were less likely to be contributed by the test itself. This represents the current practical limitation of the test-guided approach, where benefit is limited in critically-ill patients with poor short-term survival due to the time delay to test initiation. A randomised controlled study is currently in progress to establish the differences between test-guided and conventional approaches and to validate the findings in this study. Nevertheless, this is one of the biggest real-world cohorts investigating the in vitro bactericidal activity of an extensive number of combinations against diverse CRPAs, potentially informing empiric treatment of CRPAs. The clinical outcomes of patients with varying disease severity and infection types receiving these bactericidal combinations were prospectively followed up, supporting the feasibility and safety of utilising personalised in vitro test-guided treatment of CRPA infections.

Secondly, the generalisability of the study results to settings with different CRPA molecular epidemiology or to other Gram-negative infections e.g. Enterobacterales/*Acinetobacter baumannii* may be limited. However, the results here could provide base knowledge for combination activities against CRPA with various different genotypes and resistance mechanisms that could be useful for future investigations.

In conclusion, personalised *i*ACT-guided combination antibiotic treatment achieved positive clinical and microbiological outcomes in our patients, resulting in high response rates and corresponding low mortality. The current study demonstrated the potential of *i*ACT-guided therapy through the selection of bactericidal antimicrobial combinations coupled with individualised dose optimisation using pharmacokinetic/pharmacodynamic principles in combating infections caused by CRPA, even in difficult-to-treat MBL-producing isolates such as the locally circulating ST308 clones. Where *i*ACT is not available, polymyxin combinations are the most reliable in exhibiting bactericidal activity. Polymyxin-sparing regimens, like aztreonam or fosfomycin combinations, are useful to prevent polymyxin-related adverse side-effects if prolonged duration of therapy is expected. The findings in this study should be confirmed with subsequent controlled studies.

## Supplementary information

Supplementary information

Description of Additional Supplementary Files

Supplementary Data 1-4

Reporting summary

## Data Availability

Individual-level data used for this study are not openly available due to reasons of sensitivity and data privacy regulations. Access to the data used for this study can be made available upon reasonable request to the corresponding author (andrea.kwa.l.h@sgh.com.sg), subject to institutional approval and completion of a data sharing agreement and in accordance with the prevailing data sharing guidelines of SGH. All aggregated-level data supporting the findings of this study are available within the paper and its Supplementary Information. Whole-genome sequences used in this study are available in the NCBI Sequence Read Archive under BioProject accession number PRJNA656645 (accession numbers are available in Supplementary data 1).
