# Supplementary material for: Personalised bactericidal combination regimens against carbapenem-resistant Pseudomonas aeruginosa
Source: Commun Med (Lond). 2025 Aug 5;5:334. doi: 10.1038/s43856-025-01022-2 (PMC12325961; doi:10.1038/s43856-025-01022-2)
Supplement: Supplementary file 1 — Supplementary information [file 43856_2025_1022_MOESM1_ESM.pdf]

Supplementary figure 1. Workflow depicting the *in vitro* antibiotic combination test clinical service

In vitro antibiotic combination test (*i*ACT) clinical service

Patient with resistant infection

LABORATORY	DAY	BEDSIDE
After the isolate is received at the pharmacy laboratory, the test is performed using 96-well plates containing >160 combinations at clinically relevant concentrations.	0	Clinical case notes are reviewed by the specialist infectious disease (ID) pharmacist. Upon case discussion with the ID physician, the empiric regimen is optimised, if necessary.
After 24h incubation, the plates are inspected visually and non-turbid wells (combinations which are at least inhibitory) are plated out.	1	The plate results are interpreted by the ID pharmacist to ensure that the empiric regimen is at least inhibitory.
The incubated plates are enumerated and evaluated for bactericidal activity (≥3 log decrease from starting inoculum).	2	Bactericidal combinations are identified by the ID pharmacist. Suitable combinations individualised (including dosing) in accordance to infection source and individual patient characteristics and considerations. Recommendations are made to the ID physician through case discussion and documented in a report.

Therapy implementation and patient monitoring

Supplementary figure 2. Sample iACT report

Accession Number	Name	Age	Weight	NRIC/FIN No.	Organism	Source	Location
[redacted]	[redacted]	70	67.5	[redacted]	*Pseudomonas aeruginosa*	Blood	SGH

Microbroth MIC (mg/L)											
LVX	FEP	IPM	MEM	DOR	ATM	TZP	PMB	GEN	C/T	AMK	CZA
>=64	>=256	>=64	>=64	>=64	64	64/4	2	>=64	>=128/4	>=256	>=128/4

Etest MIC (mg/L)	
Rifampicin	Fosfomycin
>=64	48

ANTIMICROBIALS

LVX	Levofloxacin	TZP	Piperacillin / Tazobactam constant 4
FEP	Cefepime	PMB	Polymyxin B
IPM	Imipenem	GEN	Gentamicin
MEM	Meropenem	C/T	Ceftolozane / Tazobactam constant 4
DOR	Doripenem	AMK	Amikacin
ATM	Aztreonam	CZA	Ceftazidime / Avibactam constant 4

Remarks:

Mdm [redacted]s a patient with a complicated history of recurrent UTI. She has now developed MDRPA bacteraemia post elective right URS.

Based on the combination testing results, we recommend the following regimens:

1. Aztreonam 2g loading dose, followed by 2g infused over 4h q6H + piperacillin/tazobactam 4.5g loading dose followed by 4.5g infused over 4h q6H <u>OR</u>

2. Aztreonam 2g loading dose, followed by 2g infused over 4h q6H + IV/PO levofloxacin 500mg q12H <u>OR</u>

3. Piperacillin/tazobactam 4.5g loading dose followed by 4.5g infused over 4h q6H + IV/PO levofloxacin 500mg q12H

Should the patient deteriorate/spike fever, IV Amikacin 1250mg q24H may be added on for 3 - 5 days.

The doses are recommended in view of the patient's high urine output (>4L daily). Should there be fluctuations in the patient's renal function or the need to select a regimen for OPAT, please contact one of the ID pharmacists in-charge.

Please call us if needed.

Please note that administration of lower doses than the above suggestions will result in inadequate concentrations of drugs at the infected sites and can render these supposedly effective combinations ineffective.
