# Supplementary material for: Personalised bactericidal combination regimens against carbapenem-resistant Pseudomonas aeruginosa
Source: Commun Med (Lond). 2025 Aug 5;5:334. doi: 10.1038/s43856-025-01022-2 (PMC12325961; doi:10.1038/s43856-025-01022-2)
Supplement: Supplementary file 2 — Description of Additional Supplementary Files [file 43856_2025_1022_MOESM2_ESM.docx]

**Description of Additional Supplementary Files**

File name: Supplementary Dataset

Description:

1. Supplementary data 1 - Phenotypic and genotypic characteristics of 66 CRPA isolates
2. Supplementary data 2 - Cases excluded from outcomes analyses
3. Supplementary data 3 - Treatment and outcomes of 42 clinically evaluable cases
4. Supplementary data 4 - Cases requiring further therapy modification post-*i*ACT
