# Supplementary material for: Personalised bactericidal combination regimens against carbapenem-resistant Pseudomonas aeruginosa
Source: Commun Med (Lond). 2025 Aug 5;5:334. doi: 10.1038/s43856-025-01022-2 (PMC12325961; doi:10.1038/s43856-025-01022-2)
Supplement: Supplementary file 4 — Reporting summary [file 43856_2025_1022_MOESM4_ESM.pdf]

Corresponding author(s): Andrea Kwa

Last updated by author(s): Jun 2, 2025

Reporting Summary

Nature Portfolio wishes to improve the reproducibility of the work that we publish. This form provides structure for consistency and transparency in reporting. For further information on Nature Portfolio policies, see our Editorial Policies and the Editorial Policy Checklist.

Statistics

For all statistical analyses, confirm that the following items are present in the figure legend, table legend, main text, or Methods section.

n/a	Confirmed	
☒	☐	The exact sample size (*n*) for each experimental group/condition, given as a discrete number and unit of measurement
☒	☐	A statement on whether measurements were taken from distinct samples or whether the same sample was measured repeatedly
☒	☐	The statistical test(s) used AND whether they are one- or two-sided<br>*Only common tests should be described solely by name; describe more complex techniques in the Methods section.*
☒	☐	A description of all covariates tested
☒	☐	A description of any assumptions or corrections, such as tests of normality and adjustment for multiple comparisons
☒	☐	A full description of the statistical parameters including central tendency (e.g. means) or other basic estimates (e.g. regression coefficient) AND variation (e.g. standard deviation) or associated estimates of uncertainty (e.g. confidence intervals)
☒	☐	For null hypothesis testing, the test statistic (e.g. *F*, *t*, *r*) with confidence intervals, effect sizes, degrees of freedom and *P* value noted<br>*Give P values as exact values whenever suitable.*
☒	☐	For Bayesian analysis, information on the choice of priors and Markov chain Monte Carlo settings
☒	☐	For hierarchical and complex designs, identification of the appropriate level for tests and full reporting of outcomes
☒	☐	Estimates of effect sizes (e.g. Cohen's *d*, Pearson's *r*), indicating how they were calculated

Our web collection on statistics for biologists contains articles on many of the points above.

Software and code

Policy information about availability of computer code

Data collection: Not applicable.

Data analysis: Not applicable.

For manuscripts utilizing custom algorithms or software that are central to the research but not yet described in published literature, software must be made available to editors and reviewers. We strongly encourage code deposition in a community repository (e.g. GitHub). See the Nature Portfolio guidelines for submitting code & software for further information.

Data

Policy information about availability of data

All manuscripts must include a data availability statement. This statement should provide the following information, where applicable:

- Accession codes, unique identifiers, or web links for publicly available datasets
- A description of any restrictions on data availability
- For clinical datasets or third party data, please ensure that the statement adheres to our policy

All data supporting the findings of this study are available within the paper and its Supplementary Information.

Research involving human participants, their data, or biological material

Policy information about studies with human participants or human data. See also policy information about sex, gender (identity/presentation), and sexual orientation and race, ethnicity and racism.

Reporting on sex and gender	Study does not categorize the study population into socially relevant groups for analyses. The term "sex" is being used. Sex determined from hospital records, it was not considered in study design. Disaggregated data presented.
Reporting on race, ethnicity, or other socially relevant groupings	Not applicable.
Population characteristics	Age, years (mean ± SD): 60.5 ±14.2; Male sex: n=26 (625)<br>Age, years (mean ± SD): 60.5 ±14.2; Male sex: n=26 (62%)
Recruitment	Patients ≥21 years old admitted between October 2017 and October 2022 with positive carbapenem-resistant Pseudomonas aeruginosa (CRPA) clinical cultures were prospectively identified from the institution's microbiology laboratory. Patients suitable for in vitro antibiotic combination tests (iACTs) were referred by infectious diseases physician and tests were conducted on CRPA isolates deemed to be implicated in infections by the infectious diseases physician.
Ethics oversight	The research was approved by the SingHealth Centralised Institutional Review Board (Reference number: 2017/2047).

Note that full information on the approval of the study protocol must also be provided in the manuscript.

Field-specific reporting

Please select the one below that is the best fit for your research. If you are not sure, read the appropriate sections before making your selection.

☒ Life sciences ☐ Behavioural & social sciences ☐ Ecological, evolutionary & environmental sciences

For a reference copy of the document with all sections, see nature.com/documents/nr-reporting-summary-flat.pdf

Life sciences study design

All studies must disclose on these points even when the disclosure is negative.

Sample size	No sample size calculation was performed.
Data exclusions	Exclusion criteria was pre-determined. Our study objective was to detail the clinical characteristics, clinical course and potential impact of personalised iACT-guided therapy on patients with difficult-to-treat CRPA infections hence we have focused on the clinical evaluation of patients with clinically relevant CRPA infections and were utilising the iACT-guided treatment approach. Therefore, the following patients were excluded from the clinical evaluation portion of the study. 1. Consecutive isolates (cases already included and point of reference taken from the index culture). 2. Died prior to receipt of guided-therapy (it is unlikely the outcome of the cases were related to guided-therapy if antibiotics were received for <72h). 3. Not started on guided-therapy (these patients were not managed on active therapy as it is likely that the P. aeruginosa was not clinically significant and eventually deemed not to be implicated in the infection). 4. Pre-emptive testing (these patients were not managed on active therapy as P. aeruginosa were deemed colonisers and testing was performed pre-emptively for future scenarios when/if patient falls sick). 5. Polymicrobial infection (we excluded infections involving concomitant carbapenem-resistant organisms as this study focused specifically on carbapenem-resistant P. aeruginosa)
Replication	Not applicable.
Randomization	This is a prospective cohort study - no randomization was performed.
Blinding	This is a prospective cohort study - no blinding was performed.

Reporting for specific materials, systems and methods

We require information from authors about some types of materials, experimental systems and methods used in many studies. Here, indicate whether each material, system or method listed is relevant to your study. If you are not sure if a list item applies to your research, read the appropriate section before selecting a response.

Materials & experimental systems

n/a	Involved in the study
☒	☐ Antibodies
☒	☐ Eukaryotic cell lines
☒	☐ Palaeontology and archaeology
☒	☐ Animals and other organisms
☐	☒ Clinical data
☒	☐ Dual use research of concern
☒	☐ Plants

Methods

n/a	Involved in the study
☒	☐ ChIP-seq
☒	☐ Flow cytometry
☒	☐ MRI-based neuroimaging

Clinical data

Policy information about clinical studies

All manuscripts should comply with the ICMJE guidelines for publication of clinical research and a completed CONSORT checklist must be included with all submissions.

Clinical trial registration	Not applicable - not a RCT, this is a prospective cohort study
Study protocol	Not applicable - not a RCT, this is a prospective cohort study
Data collection	This was a single-centre prospective cohort study conducted in Singapore General Hospital (SGH), a 1800-bedded public acute care tertiary hospital. Patients ≥21 years old admitted between October 2017 and October 2022 with positive CRPA clinical cultures were prospectively identified from the institution's microbiology laboratory. In vitro antibiotic combination tests (iACTs) were conducted on CRPA isolates deemed to be implicated in infections by the attending infectious diseases (ID) physicians. The research was approved by the SingHealth Centralised Institutional Review Board (Reference number: 2017/2047). Patients' consent were sought prior to iACT commencement.
Outcomes	Patients who received iACT-guided bactericidal regimens for at least 72h from time of iACT initiation were assessed for clinical outcomes. All clinical data were collected from the electronic medical records and documented in a structured data collection form. The clinical and microbiological outcomes included 30-day all-cause mortality, 30-day infection-related mortality, end-of-therapy clinical response, end-of-therapy microbiological eradication, and adverse drug reactions (ADRs) associated with iACT-guided therapies.

Plants

Seed stocks	Not applicable.
Novel plant genotypes	Not applicable.
Authentication	Not applicable.
